# Prevalence of Malnutrition in People Hospitalized for Surgery: Prospective Cross-Sectional Study

**DOI:** 10.3390/healthcare13040380

**Published:** 2025-02-11

**Authors:** Abdulrahman Alamri, Kholoud Alaamer, Yasser Almogbel, Hanan Alsalahi, Mananl Al Shareef, Saleh Alanazi, Hamza Al Samannoudi, Fahad Alhusaini

**Affiliations:** 1 Pharmaceutical Care Services, King Abdulaziz Medical City, Ministry of the National Guard Health Affairs, Riyadh 11426, Saudi Arabia; alaamerkh@mngha.med.sa (K.A.); anazis@mngha.med.sa (S.A.); samannoudih@mngha.med.sa (H.A.S.); 2 King Abdullah International Medical Research Center, Riyadh 11481, Saudi Arabia; alsalahiha@mngha.med.sa (H.A.); alshareefma1@mngha.med.sa (M.A.S.); hussainif@mngha.med.sa (F.A.); 3 Department of Pharmacy Practice, College of Pharmacy, King Saud Bin Abdulaziz University for Health Sciences, Riyadh 11481, Saudi Arabia; 4 Department of Pharmacy Practice, College of Pharmacy, Qassim University, Buraidah 51452, Saudi Arabia; y.almogbel@qu.edu.sa; 5 Clinical Nutrition Services, Ministry of the National Guard Health Affairs, Riyadh 11426, Saudi Arabia

**Keywords:** nutritional assessment, malnutrition, prevalence of malnutrition, GLIM, MUST, MNA-SF

## Abstract

**Background/Objectives**: Malnutrition poses a significant challenge to public health, affecting millions worldwide, particularly among people in hospital, notably among surgical cases that require adequate nutritional support for effective recovery. Factors contributing to malnutrition include chronic illnesses that hinder nutritional intake and socioeconomic barriers limiting food access. This study aimed to assess malnutrition in surgical patients at admission to enhance recovery, minimize complications, and improve clinical outcomes. **Methods**: This prospective observational cross-sectional study involved 282 adults hospitalized for over 48 h for surgery. This study utilized the MUST and MNA-SF assessments to evaluate malnutrition risk, confirming diagnoses via the GLIM criteria. Statistical analysis, including the Pearson chi-square test and univariate and multivariate logistic regression, identified significant malnutrition risk factors. **Results**: This study found an 18.1% malnutrition prevalence among these patients, with Stage 1 malnutrition being the most common at 9.6%. Additionally, a notable proportion of patients were classified as obese (46.5%) or overweight (27.7%). Men had higher malnutrition rates (12.8%) than women (5.3%). Age disparities were also significant, with higher rates among older (7.4%) and younger adults (7.1%) compared to middle-aged individuals (2.5%). Patients undergoing elective surgeries (9.9%) and emergency surgeries (6.7%) had higher malnutrition rates than those admitted for surgical complications. Furthermore, malnutrition was observed in 12% of patients undergoing orthopedic or general surgeries. **Conclusions**: Among adult patients admitted to surgical wards, malnutrition prevalence during admission was 18%, underscoring the need for comprehensive preoperative nutritional assessments and targeted interventions for patients undergoing surgery.

## 1. Introduction

Malnutrition is a subacute or chronic nutritional condition characterized by alterations in body composition and diminished functionality, resulting from a combination of inflammatory activity, overnutrition, and undernutrition. Both undernutrition and overnutrition are forms of malnutrition, but underweight individuals tend to be more susceptible to health issues due to factors such as energy deficits, nutrient deficiencies, metabolic impacts, and inflammation. It is prevalent among people in hospital, affecting approximately 22% of them, and is linked to longer hospital stays and elevated mortality rates [1,2]. People admitted to hospitals for surgery often experience malnutrition, making it the third most common cause of hospital readmission following surgery [3,4].

Ljungqvist et al. [5] revealed that malnourished patients have a fourfold higher risk of death. It was estimated that 24–65% of people undergoing surgery are at risk of malnutrition [6]. Furthermore, observational data from patients undergoing elective colorectal surgery revealed that malnourished or at-risk individuals have a twofold increased likelihood of readmission within 30 days after the procedure [6]. Prolonged fasting before surgery is a primary risk factor for malnutrition in surgical patients, resulting in a catabolic metabolism, muscle degradations, a reduction in liver glycogen stores, heightened insulin resistance, and elevated blood glucose levels. Additionally, the postoperative stress response in patients undergoing surgery can further exacerbate the risk of malnutrition [7]. Furthermore, postoperative hyperglycemia has been independently correlated with increased morbidity and mortality rates [7].

Preoperative malnutrition can increase the risk of infection, delay wound healing, and prolong hospital stays [4]. In contrast, adequate preoperative nutrition can reduce complications and shorten hospital and intensive care stays [4]. Preoperative nutrition support has been shown to reduce morbidity by 20% [8,9]. A study conducted on older patients in Saudi Arabia indicated that 5.3% of participants were severely malnourished, whereas 32.9% were at risk of malnutrition [10]. Another study on older adults visiting primary care centers in Saudi Arabia revealed that 20.9% of the population was malnourished, with a strong correlation between daily living activities and nutritional status [11]. However, these studies did not include patients hospitalized for surgery.

The Mini Nutritional Assessment Short-Form (MNA-SF) and the Malnutrition Universal Screening Tool (MUST) are commonly utilized instruments for nutrition screening and assessment [12]. The MUST tool was produced to assess the risk of malnutrition in patients across all care settings, using data on body mass index (BMI), computed weight loss percentage, and food intake. The MNA-SF is frequently used for nutritional assessment in older patients, considering factors such as BMI, food intake, weight loss, mobility, and neuropsychological issues [13]. The Global Leadership Initiative on Malnutrition (GLIM) is a recently validated tool for diagnosing malnutrition, focusing on etiologic criteria and phenotypic criteria in adults [14].

A study on patients with Crohn’s disease demonstrated that using the GLIM criteria is more accurate in diagnosing malnourished patients than other clinical scores, emphasizing the importance of providing sufficient and appropriate nutrition support therapy [15]. Alternatively, applying the GLIM criteria to patients on hemodialysis may serve as a predictor of mortality and infection rates [16].

In Saudi Arabia, limited research has been conducted on the prevalence of malnutrition among hospitalized patients nationwide. In addition, malnutrition is frequently observed in surgical patients; however, the prevalence, risk factors, and outcomes of malnutrition in this population remain unexplored. This research aimed to explore the prevalence of malnutrition and identify the key factors linked to malnutrition in a tertiary hospital in Saudi Arabia among people undergoing surgery at the time of hospital admission. By understanding the underlying causes and implications of malnutrition in this specific population, we can enhance clinical practices, promote timely nutritional interventions, and ultimately improve patient outcomes. Through a comprehensive analysis, this study highlights the importance of recognizing and addressing malnutrition as an integral component of surgical care.

## 2. Materials and Methods

### 2.1. Assessment of the Nutritional Status

The evaluation of malnutrition was carried out using the standards set by the Global Leadership Initiative on Malnutrition, coordinated by various leading global clinical nutrition organizations. This process involves a two-step approach for diagnosing malnutrition: first, screening to determine “at risk” status using any validated screening tool; second, assessing the diagnosis and categorization of the severity of malnutrition [14].

The GLIM criteria include three physical indicators (phenotypic criteria): notable weight loss, low body mass index (BMI), and decreased muscle mass. We also took into account two causative factors (etiologic criteria): reduced food intake or absorption and heightened disease burden or inflammation (Table 1 and Table 2). Malnutrition was diagnosed when a patient satisfied at least one phenotypic and one etiologic criterion. Additionally, to determine the severity of malnutrition, meeting just one phenotypic criterion was enough to classify it as Stage 1 (moderate) or Stage 2 (severe). This thorough method guaranteed a clear and organized assessment of malnutrition in our patients [14].

### 2.2. Sample Size

A sample size of 277 was calculated using a single proportion equation with the “Raosoft software package” (http://www.raosoft.com/info/pricing/packages.html, accessed on 7 February 2025), with an absolute precision of 5% and a confidence level of 95%. The anticipated frequency was based on a reference article [17]. Due to a 100% response rate, the estimated sample size was increased by 0–2% to 282. All patients who met the inclusion/exclusion criteria during the study period were included in this evaluation.

### 2.3. Study Design and Participants

A prospective observational cross-sectional study was conducted, involving 282 patients admitted to the surgery wards at King Abdulaziz Medical City, Ministry of National Guard Health Affairs in Riyadh, Saudi Arabia, between 1 February 2023 and 30 July 2023. This tertiary care hospital, with 1973 operational beds, offers a wide range of healthcare services, including public health, primary care, specialized treatments, and tertiary services. King Abdulaziz Medical City houses specialized centers like an organ transplant center, a trauma and surgical center, a cardiovascular center, and an oncology center. It is also home to the King Abdullah Specialized Children’s Hospital, a dedicated referral center for pediatric care. The facility employs 5282 administrative and support professionals, 8584 employees including allied health and medical support professionals, as well as 2451 physicians, dentists, and residents.

### 2.4. Inclusion Criteria

All adult patients aged 18 years or older who were admitted to surgical wards for more than 48 h were included in this study.

### 2.5. Exclusion Criteria

Patients under the age of 18, pregnant, unable or unwilling to provide written informed consent, or hospitalized for less than 48 h were excluded from this study.

### 2.6. Data Collection Procedures, Data Sources, and Measurements

Clinical data were collected through patient interviews and a review of electronic medical records using the Health Information System BESTCare^®^, Seoul, Republic of Korea. Patient information was systematically recorded using an approved data collection form. The collected data included demographic details, admission department, reasons for admission, diagnoses, underlying health conditions, and reductions in food intake. Reduced muscle mass was assessed through physical examination or by measuring the mid-arm muscle circumference (MAMC), calculated using the standard formula: MAMC = MUAC − (0.314 × triceps skinfold) in mm.

### 2.7. Statistical Analysis

Data analysis was performed using the Statistical Package for the Social Sciences version 24. Descriptive statistics were calculated for both numerical and categorical variables, including means and standard deviations for continuous variables, as well as frequencies and percentages for categorical variables. The Pearson chi-square test assessed the distribution of malnutrition (based on the GLIM criteria) across various demographic groups and factors. If the assumptions of the Pearson chi-square test were not satisfied, Fisher’s exact test was utilized. Univariate and multivariate logistic regression analyses were conducted to identify significant risk factors for malnutrition in hospitalized patients. Variables with a significance level of *p* < 0.20 in the univariate analysis were included in a combined multivariate model to evaluate their influence on the likelihood of malnutrition in hospitalized patients [18]. A significance level of *p* < 0.05 was deemed statistically significant.

### 2.8. Ethical Consideration

This study was approved by the King Abdullah International Medical Research Center Institutional Review Board in Riyadh, Saudi Arabia (Ref. IRB/0436/23 on 12 February 2023). Informed consent was obtained from all participants, and strict confidentiality measures were implemented to ensure the protection of research data and participant information, which were used exclusively for research purposes. The investigations were performed following the criteria of the Declaration of Helsinki of 1975, which was revised in 2013.

## 3. Results

In this study, 282 hospitalized patients were included, with women comprising the majority (*n* = 143, 50.7%) compared to men (*n* = 139, 49.3%). The mean age of the participants was 55.76 ± 18.67 years, ranging from 18 to 95 years. The mean BMI in women was higher at 32.18 ± 6.75 than in men, who had a mean BMI of 26.91 ± 7.64. The overall prevalence of malnutrition among the hospitalized patients was 18.1%, with the highest prevalence according to the GLIM criteria observed in Stage 1 (9.6%), followed by Stage 2 (8.5%). In terms of BMI, the majority of hospitalized patients were classified as obese (46.5%) or overweight (27.7%). Approximately 11.3% of the hospitalized patients reported allergies to certain foods or drugs. Most patients (57.8%) were admitted for diagnostic purposes or elective future surgeries, with orthopedic surgeries being the most common (42.9%), followed by general surgeries (35.5%) (Table 3).

The demographic distribution of malnutrition is outlined in Table 3. Notably, malnutrition was significantly more prevalent among men (12.8%) than women (5.3%). A significant correlation was observed between malnutrition and age group, with 7.4% of older adults (aged 60–79 years) and 7.1% of young adults (aged 18–39 years) showing a higher prevalence of malnutrition compared to the middle-aged adult group (2.5%).

The distribution of malnutrition across BMI groups also revealed significant associations: individuals with normal weight (7.1%) and underweight (5.7%) were more susceptible to malnutrition than individuals with overweight (2.8%) and obesity (2.5%). Furthermore, malnutrition was linked to the reasons for admission and types of surgeries, with higher percentages among patients admitted for diagnosis or elective surgeries (9.9%) and emergency surgeries (6.7%) compared with those admitted with surgical complications. Additionally, 12% of cases of malnourishment were recorded in patients undergoing orthopedic or general surgeries, surpassing other types of surgeries in prevalence.

In terms of the distribution of underlying diseases, malnutrition was more prevalent among patients undergoing acute surgical treatment without infectious symptoms (12.1%) compared to those with infectious symptoms (4.3%) and trauma-related cases (1.8%), with no statistical significance. Conversely, patients hospitalized with chronic diseases related to the endocrine system (7.4%) and cardiovascular system (7.4%) exhibited a significantly higher susceptibility to malnutrition than the other chronic disease groups (Table 4).

Advanced univariate logistic regression was used to identify the significant risk factors for malnutrition, with the results shown in Table 5.

The odds of malnutrition status were 0.33 times lower among women than among men (odds ratio [OR]: 2.983, 95% confidence interval [CI]: 1.54–5.74). Hospitalized patients with diabetes had 0.487 times lower odds of malnutrition (OR: 0.487, 95% CI: 0.26–0.91) than patients without diabetes. Similarly, patients with hypertension were 0.487 times less likely to experience malnutrition (OR: 0.487, 95% CI: 0.25–0.92) than patients without hypertension. Significant factors (*p* < 0.20) were included in a multivariate model [18] to assess their impact on malnutrition risk in the hospitalized patients. Every unit increase in BMI was associated with a 0.256 decrease in the log odds of malnutrition among the hospitalized patients (OR: 0.774, 95% CI: 0.71–0.84, *p* < 0.001). Patients with ischemic heart disease (percutaneous coronary intervention or surgically treated coronary artery bypass graft) were 4.836 times more likely to experience malnutrition than patients with non-ischemic heart disease (95% CI: 1.46–16.01, *p* = 0.010). In the univariate logistic regression analysis, age and chronic diseases such as diabetes and hypertension, which were significant, became insignificant in the multivariate logistic regression analysis when other covariates were considered.

## 4. Discussion

### 4.1. Prevalence

Our study revealed that 18.1% of the patients admitted to hospital surgical departments were malnourished at the time of admission, as verified by the Global Leadership Initiative on Malnutrition (GLIM) criteria. This prevalence aligns with a spectrum of previously reported rates in similar settings, which have varied significantly, ranging from 1.5% to 29% [10,11,17]. A cross-sectional study conducted at a tertiary outpatient clinic in Saudi Arabia utilized the Mini Nutritional Assessment Short Form (MNA-SF) and found a lower malnutrition rate of 5.3%, with 32.9% of participants identified as being at risk of malnutrition [10]. Similarly, a larger study at primary healthcare centers involving 2045 older patients reported a malnutrition prevalence of just 1.5%, alongside a 19.4% risk of malnutrition [11].

In contrast, an investigation involving 248 hospitalized older patients in Saudi Arabia revealed a higher rate of 29% malnutrition using the MNA-SF tool (Bafakeeh et al., 2020) [17]. Another cross-sectional study conducted in Lebanon, employing the Nutritional Risk Screening Tool 2002 alongside GLIM criteria, included hospitalized patients from random hospitals in five districts to assess malnutrition prevalence, finding an alarming 35.6% prevalence of malnutrition among hospitalized patients [19].

Furthermore, a multicenter cross-sectional study in Korea, assessing nutritional status via the Subjective Global Assessment across 300 patients from 25 hospitals, noted a malnutrition prevalence of 22.0% [2]. These findings underscore the public health concern of malnutrition in hospitalized patients and highlight the need for consistent screening practices across diverse healthcare settings.

### 4.2. Age

Malnutrition is a critical public health issue that disproportionately affects vulnerable populations, particularly individuals across various age groups. Our study revealed a significant correlation between age and malnutrition prevalence, with patients aged 60 years and older exhibiting a malnutrition rate of 7.4%. This contrasts sharply with the 2.5% rate observed in middle-aged adults (ages 40–59 years) and a notable 7.1% prevalence among young adults (ages 18–39 years), highlighting the complex interplay of age and nutritional status (*p* = 0.006).

These rates are consistent with previous studies, underscoring the importance of age as a determinant of malnutrition. For instance, a study conducted in Saudi Arabia documented a malnutrition prevalence of 5.3% among the geriatric population, which supports our observation of heightened malnutrition rates in older adults [10].

Moreover, several scholarly articles emphasize the necessity for routine nutritional assessments across all age groups but particularly for older adults where malnutrition can exacerbate health conditions and prolong recovery times [20]. One systematic review noted that older adults frequently encounter barriers to proper nutrition, including physiological changes related to aging and decreased appetite [21]. Given the high prevalence of malnutrition in the elderly, regular and systematic nutritional assessments should be conducted upon admission and during hospitalization to ensure proper nutritional intervention [22,23,24].

Conversely, a European study conducted in Germany found a malnutrition prevalence of 7.8% in patients under 30 years old, suggesting that young adults may also be at heightened risk for nutritional deficiencies and highlights the unexpected but critical malnutrition concerns in younger populations [22]. These data are critical as they challenge the common misconception that malnutrition is solely a concern for older adults.

### 4.3. Sex

Our research findings revealed a notable discrepancy in the prevalence of malnutrition between the sexes, with men exhibiting a significantly higher rate of 12.8% compared to only 5.3% in women (*p* = 0.001). This outcome diverges from the results of previous studies that have reported contrasting figures regarding malnutrition rates among the sexes. In Saudi Arabia, a study that focused on older outpatient populations found that women had a significantly higher rate of malnutrition at 44.6% compared to 28.3% in men (*p* = 0.044) [10]. Another study conducted in primary healthcare centers with 2045 older patients also showed a higher prevalence of malnutrition or being at risk of malnutrition in older women (24.9%) than in older men (17.7%) [11]. A study by Smith et al. (2020) indicated that malnutrition was more prevalent among females, demonstrating the complexity and variability of this public health issue [25]. One potential explanation for the observed differences in our study may lie in the mean body mass index (BMI) values recorded for each sex. The mean BMI for women was notably higher at 32.18 ± 6.75, while men had a mean BMI of 26.91 ± 7.64. The elevated BMI among women could contribute to a lower prevalence of malnutrition, as higher BMI levels are often associated with better nutritional status.

However, globally, malnutrition affects both men and women, but the prevalence can vary by region, age, group and socioeconomic factors. Historically, women and girls especially in developing countries have faced higher rates of malnutrition due to factors like limited access to food education and healthcare. However, men may also experience malnutrition in situations of conflict or poverty. Future studies must account for these distinctions to develop effective interventions aimed at alleviating malnutrition across different populations.

### 4.4. Underlying Conditions

Our study identified specific underlying comorbidities, particularly in patients with chronic endocrine and cardiovascular diseases, that were linked to malnutrition. Although we did not find a significant association between diabetes and malnutrition in our study, prior research suggests a substantial prevalence of malnutrition among patients with diabetes, who may be at risk of postoperative complications and reduced quality of life [26,27,28]. A meta-analysis of 30 studies involving 37,303 patients with acute coronary syndrome reported a malnutrition rate of 33.5% and an overall mortality rate of 20.59%, highlighting the strong link between malnutrition and all-cause mortality following acute coronary syndrome [29]. Furthermore, a review of 30 cohort studies with 81,361 participants with coronary artery disease demonstrated that malnutrition was independently linked to a 72% higher risk of all-cause mortality, particularly among patients with stable coronary artery disease [30]. These findings emphasize the necessity of implementing screening and intervention strategies for hospitalized patients with malnutrition [31].

### 4.5. Reasons for Admission and Type of Surgery

Our study revealed a higher prevalence of malnutrition among patients admitted for diagnostic or elective surgeries (9.9%) compared with those undergoing emergency surgeries (6.7%) or surgical complications (1.4%). Notably, 12% of malnourishment cases were observed in patients undergoing orthopedic or general surgeries, which was higher than in the other surgical categories. Several reports support our findings, indicating that malnutrition rates are higher among patients admitted for diagnostic procedures or elective surgeries than those dealing with surgical complications, particularly in gastrointestinal and oncologic cases, where over 60% of patients are malnourished before surgery [32,33]. Additionally, a single-center observational study involving 467 patients assessed the prevalence of malnutrition risk in individuals referred to a preanesthetic clinic, focusing on consecutive patients scheduled for elective surgical procedures. Participants were categorized based on the GLIM criteria, with 19.9% falling into the category of malnutrition risk and 7.9% meeting the criteria for preoperative nutritional support [34].

All of the previous evidence underscores the importance of optimizing nutritional status preoperatively to enhance recovery and reduce postoperative morbidity and mortality rates [35,36,37,38].

This study has several limitations. First, the nutritional status was assessed only upon admission, excluding patients who were already hospitalized, possibly underestimating the overall malnutrition rate. Second, the outcomes related to nutritional status were not adjusted for the severity of underlying conditions, which may have led to bias. This nationwide prospective study in Saudi Arabia is the first to assess hospital inpatient malnutrition rates using the GLIM criteria, which can help enhance hospital nutritional support programs. However, the small sample size and heterogeneity of this study group, with diverse diseases and stages, could impact the results.

## 5. Conclusions

This study highlights a notable prevalence of malnutrition among adult patients admitted to surgical wards in a large tertiary hospital in Saudi Arabia, recorded at 18%. The findings indicate particularly elevated rates among patients aged over 60 years and those under 40 years, with a significant disparity favoring men over women. Additionally, malnutrition was more prevalent among patients undergoing diagnostic or elective procedures and those with chronic endocrine and cardiovascular diseases. These results underscore the critical need for comprehensive preadmission nutritional assessments and the timely implementation of targeted nutritional interventions for patients undergoing surgery.

## Figures and Tables

**Table 1:** Phenotypic and etiologic criteria for the diagnosis of malnutrition.

Phenotypic Criteria			Etiologic Criteria	
Weight Loss (%)	Low Body Mass Index (kg/m^2^)	Reduced Muscle Mass	Reduced Food Intake or Assimilation	Inflammation
>5% within past 6 months, or >10% beyond 6 months	<20 if <70 years, or <22 if >70 years	Reduced by validated body-composition-measuring techniques	≤50% of ER > 1 week or any reduction for >2 weeks or any chronic GI condition that adversely impacts food assimilation or absorption	Acute disease/injury or chronic disease-related

**Table 2:** Grade of malnutrition severity.

Stage	Phenotypic Criteria		
	Weight Loss (%)	Low BMI Index (kg/m^2^)	Reduced Muscle Mass
Stage 1 moderate malnutrition (Need 1 phenotypic criterion that meets this grade)	5–10% in the last 6 months, or 10–20% beyond 6 months	<20 if <70 years, <22 if ≥70 years	Mild to moderate deficit (via validated assessment methods)
Stage 2 severe malnutrition. (Need 1 phenotypic criterion that meets this grade)	>10% within the past 6 mo, or >20% beyond 6 mo	<18.5 if <70 years, <20 if ≥70 years	Severe deficit (via validated assessment methods—see below)

**Table 3:** Demographic characteristics of the hospitalized patients according to malnutrition (GLIM criteria).

Characteristics	*n* = 282 (%)	Malnutrition Absent *n* = 231 (%)	Malnutrition Present *n* = 51 (%)	*p*-Value
Sex				
Male	139 (49.3)	103 (36.5)	36 (12.8)	0.001 ^α^
Female	143 (50.7)	128 (45.4)	15 (5.3)	
Age				
18–39 years	62 (22.0)	42 (14.9)	20 (7.1)	
40–59 years	73 (25.9)	66 (23.4)	7 (2.5)	0.006 ^α^
60–79 years	128 (45.4)	107 (37.9)	21 (7.4)	
80–95 years	19 (6.7)	16 (5.7)	3 (1.1)	
BMI				
Underweight	143 (50.7)	1 (0.4)	16 (5.7)	
Normal	56 (19.9)	36 (12.8)	20 (7.1)	<0.001 ^α^
Overweight	78 (27.7)	70 (24.8)	8 (2.8)	
Obese	131 (46.5)	124 (44.0)	7 (2.5)	
Allergy ^1^				
Absent	250 (88.7)	204 (72.3)	46 (16.3)	0.701 ^α^
Present	32 (11.3)	27 (9.6)	5 (1.5)	
Admission reason				
Diagnosis or elective surgery	163 (57.8)	135 (47.9)	28 (9.9)	0.025 ^α^
Emergency surgery	112 (39.7)	93 (33.0)	19 (6.7)	
Surgical complication	7 (2.5)	3 (1.1)	4 (1.4)	
Surgery type				
General surgery	100 (35.5)	83 (29.4)	17 (6.0)	
Orthopedic surgery	121 (42.9)	104 (36.9)	17 (6.0)	0.038 ^β^
Urological surgery	21 (7.4)	12 (4.3)	9 (3.2)	
Neurosurgery	14 (5.0)	10 (3.5)	4 (1.4)	
Plastic surgery	10 (3.5)	9 (3.2)	1 (0.4)	
Thoracic surgery	4 (1.4)	2 (0.7)	2 (0.7)	
Vascular surgery	10 (3.5)	9 (3.2)	1 (0.4)	
OMS ^2^	2 (0.7)	2 (0.7)	0 (0.0)	

^α^ Pearson chi-square test, ^β^ Fisher’s exact test, ^1^ allergy, food/drugs, ^2^ OMS, oral maxillofacial surgery.

**Table 4:** Underlying diseases of hospitalized patients according to malnutrition (GLIM criteria).

Characteristics	*n* = 282 (%)	Malnutrition Absent *n* = 231 (%)	Malnutrition Present *n* = 51 (%)	*p*-Value
Surgically treated acute cases				
Infectious	56 (19.9)	44 (15.6)	12 (4.3)	
Non-infectious	180 (63.8)	146 (51.8)	34 (12.1)	0.347 ^α^
Trauma and major factures	46 (16.3)	41 (14.5)	5 (1.8)	
Chronic diseases (yes)				
Endocrinal disease	153 (54.3)	132 (46.8)	21 (7.4)	0.038 ^α^
Cardiovascular disease	161 (57.1)	140 (49.6)	21 (7.4)	0.011 ^α^
Renal disease	40 (14.2)	31 (11.0)	9 (3.2)	0.434 ^α^
Gastrointestinal disease	3 (1.1)	2 (0.7)	1 (0.4)	0.452 ^β^
Neurological disease	21 (7.4)	17 (6.0)	4 (1.4)	0.548 ^β^
Pulmonary disease	23 (8.2)	20 (7.1)	3 (1.1)	0.777 ^β^
Hepatic disease	4 (1.4)	3 (1.1)	1 (0.4)	0.552 ^β^
Oncological disease	37 (13.1)	29 (10.3)	8 (2.8)	0.549 ^α^
Organ transplant	7 (2.5)	7 (2.5)	0 (0)	0.358 ^β^

^α^ Pearson’s chi-square test, ^β^ Fisher’s exact test; chronic disease (yes) category reported only.

**Table 5:** Binary logistic regression with malnutrition.

Item	Univariate			Multivariate		
	*p*-Value	OR	95% CI	*p*-Value	aOR	95% CI
Sex (female)	0.001	0.335	0.17–0.64	0.894	0.948	0.43–2.07
Age, years	0.082	0.986	0.97–1.002	0.774	1.004	0.98–1.02
BMI, kg/m^2^	<0.001	0.782	0.72–0.84	<0.001	0.774	0.71–0.84
Common chronic diseases ^3^						
Diabetes	0.025	0.487	0.26–0.91	0.646	0.803	0.31–2.05
Hypertension	0.027	0.487	0.25–0.92	0.412	0.664	0.25–1.76
IHD (PCI/CABG)	0.183	1.883	0.74–4.77	0.010	4.836	1.46–16.01
Hypothyroidism	0.528	0.701	0.23–2.11			
BPH	0.303	1.881	0.56–6.25			
CHF	0.233	2.099	0.62–7.10			
Ischemic stroke	0.625	0.684	0.15–3.13			

OR, odds ratio; aOR, adjusted odds ratio; BMI, body mass index; ^3^ common chronic disease (yes) group reported only and (no) group used as a reference, sex (male) group used as a reference; CHF, congestive heart failure; IHD, ischemic heart disease; PCI, percutaneous coronary intervention; CABG, coronary artery bypass; BPH, benign prostrate hyperplasia; *p*-value < 0.20 was significant for univariate logistic regression analysis; *p*-value < 0.05 was significant for multivariate regression analysis.

## Data Availability

The data presented in this study are available on request from the corresponding author (the data are not publicly available due to privacy or ethical restrictions).

## Abbreviations

The following abbreviations are used in this manuscript:

GLIM	Global Leadership Initiative on Malnutrition Statement
MUST	Malnutrition Universal Screening Tool
MNA_SF	Mini Nutritional Assessment—Short Form
OMS	Oral maxillofacial surgery
BMI	Body mass index
CHF	Congestive heart failure
IHD	Ischemic heart disease
PCI	Percutaneous coronary intervention
CABG	Coronary artery bypass
BPH	Benign prostrate hyperplasia
